# Impact of Burnout on Anaesthesiologists

**DOI:** 10.4274/TJAR.2024.241565

**Published:** 2024-05-03

**Authors:** Joana Berger-Estilita, Dominic Salvisberg, Ekin Köselerli, Stefan Haupt, Başak Ceyda Meço

**Affiliations:** 1 Hirslanden Medical Group Salem Spital, Institute of Anaesthesiology and Intensive Care, Bern, Switzerland; 2 University of Bern, Institute for Medical Education, Bern, Switzerland; 3 University of Porto Faculty of Medicine, Centre for Health Technology and Services Research, Porto, Portugal; 4 University of Bern, Bern, Switzerland; 5 Ankara University Faculty of Medicine, İbn-i Sina Hospital, Department of Anaesthesiology and Intensive Care Unit, Ankara, Turkey; 6 Ankara University Brain Research Center (AÜBAUM), Ankara, Turkey

**Keywords:** Anaesthesiology, occupational health physicians, professional burnout

## Abstract

Professional burnout syndrome (PBS) is an issue affecting individuals and organizations alike, characterized by emotional exhaustion and reduced effectiveness resulting from overwhelming work demands. Root causes include excessive workload, unrealistic expectations, and blurred work-life boundaries, which are often intensified by organizational culture and inadequate support systems. The consequences range from decreased productivity and creativity to high turnover rates and financial strain on organizations. Mitigating PBS requires a comprehensive approach that addresses both individual and organizational levels. Individually, stress management techniques and self-care practices are crucial for building resilience and coping with work-related stressors. Organizations play a vital role in promoting employee well-being by fostering a supportive work environment, promoting work-life balance and providing access to support systems such as counseling and mentorship programs. Leadership is key in creating a culture that values employee health and prioritizes open communication and empathy. Policy interventions can further support efforts to combat PBS by enforcing labor laws that protect employee rights, such as setting limits on working hours and ensuring access to mental health services. Additionally, incentivise organizations to prioritize employee well-being through tax incentives or certification programs can encourage proactive measures against burnout. The aim of this review is to provide a comprehensive exploration of PBS, examining its causes, consequences, and potential mitigation strategies in individuals and organizations, with a focus on anaesthesiology.

**Main Points**

• Anaesthesiologists face significant burnout, which is exacerbated by the high-stress nature of their work, long hours, and critical decision-making responsibilities, which can lead to exhaustion, depersonalization, and reduced personal accomplishment.

• Burnout among anaesthesiologists not only impacts their own mental and physical well-being but also poses risks to patient safety and quality of care, including increased medical errors, reduced empathy, and diminished patient satisfaction.

• Psychological treatments such as cognitive behavioral therapy and acceptance and commitment therapy show promise in managing burnout symptoms among anaesthesiologists, while organization-directed interventions are essential for addressing systemic factors contributing to burnout.

• Addressing burnout among anaesthesiologists requires multifaceted interventions, including promoting work-life balance, providing access to confidential mental health support, and destigmatizing help-seeking behaviors within healthcare systems.

• Despite the challenges associated with treating physician burnout, acknowledging vulnerabilities, prioritizing self-care and advocating for systemic changes within healthcare organizations are crucial steps toward cultivating a healthier, more resilient healthcare workforce.

## Introduction

While burnout syndrome received significant media coverage in the past, it has become less prominent in recent years.^1^ However, its relevance has increased as more than half of healthcare professionals report experiencing symptoms of burnout.^2^ The coronavirus disease-2019 pandemic appears to have further exacerbated this issue,^2,3^ According to a recent survey conducted by the Association of Swiss Assistant and Senior Physicians, approximately one out of every two respondents admitted feeling overwhelmed and reaching a point where they «couldn’t handle any more».^4^ These participants often expressed feelings of fatigue, exhaustion, and emotional drainedness. They also shared incidents where patient safety was compromised due to work-related fatigue, an observation supported by a recently published study that found that doctors with burnout were twice as likely to be involved in patient safety incidents.^5^

Among medical specialties, anaesthesiology seems particularly susceptible to burnout.^3,6^ The increasing awareness of mental health issues within the healthcare profession, specifically among anaesthesiologists, has sparked a demand for a deeper understanding and proactive measures. Anaesthesiologists, who play a critical role in patient care, often operate in high-pressure environments with long working hours and the need to make life-or-death decisions. Additionally, evidence shows that the risk of developing mental illnesses, such as depression or suicide, seems to be higher among anaesthetists.^7^ This review aims to delve into the various aspects of burnout within anaesthesia, its consequences, and potential methods for mitigating it.

### Occupational Hazards in Anaesthesiology

Anaesthesiologists play a multifaceted role beyond operating rooms and intensive care units. They are called upon to provide anaesthesia services in various settings, including remote locations, pre-interventional consultations, pain clinics, magnetic resonance imaging suites, and radiotherapy centers. Anaesthesiologists also play a crucial role in trauma and disaster management teams, exposing them to a range of health hazards. Even a seemingly innocuous needle prick from an unidentified source can trigger intense anxiety and fear.^8,9^

Considering these challenges, prioritizing occupational health and safety becomes imperative for anaesthesiologists. The World Health Organization (WHO) defines occupational health as emphasizing promoting and maintaining the highest physical, mental, and social well-being levels for workers across all professions (www.who.int/health-topics/occupational-health). This includes preventing work-related health issues, protecting workers from adverse health risks during employment, and creating an occupational environment that aligns with the physiological and psychological capacities of the workforce. Health hazards encountered by anaesthesiologists can be broadly categorized according to Table 1.

### Understanding Professional Burnout Syndrome (PBS)

Many individuals in our performance-driven society are struggling because of the increasing demands placed upon them. Those who are unable to manage the excessive workload are endangering both their emotional and physical health as well as their social life with family and friendships.^10,11,12^ This state of work-related stress is commonly referred to as burnout^13^.

According to the WHO, burnout is a syndrome resulting from workplace stress that should be adequately addressed.^14^ It should be noted that burnout is considered a work-related phenomenon and is not classified as a medical condition. However, it is a diagnosis listed in the 11^th^ Revision of the International Classification of Diseases.^15^ It is work-specific, occurs in individuals without any pre-existing psychopathology, and is commonly found in caregiving professions.^16^ Burnout refers specifically to phenomena in the occupational context and should not be applied to describe experiences in other areas of life.^17^

Although there are no specific criteria for burnout, it frequently leads to the onset or worsening of mental disorders such as depression, substance abuse issues, or adjustment disorders. Additionally, burnout is associated with conditions such as cardiometabolic disorders (e.g., obesity, diabetes), hypertension, lipid metabolism issues, coronary heart disease, and even increased mortality risk. Therefore, burnout can be considered a health-threatening risk condition.

The symptoms of burnout typically manifest across three dimensions: exhaustion (feeling drained and overwhelmed), depersonalization (developing cynicism or detachment toward others) and reduced personal accomplishment (experiencing diminished productivity or effectiveness) (Table 2).^1,3,15^ Unfortunately, this phenomenon is increasingly prevalent in healthcare.

### Why Anaesthesiologists?

Many studies report high levels of burnout in doctors, with psychological morbidity ranging from 19% to 47%,^18^ compared with a rate of around 18% for the general employed population.^19^ For primary care doctors or general practitioners, most studies report a moderate degree of burnout, especially for the emotional exhaustion dimension^20,21^. Anaesthesiologists also have moderate degrees of burnout, with high job satisfaction moderating the negative effects of stressors at work.^6,22,23^ However, the literature is inconsistent in what medical speciality has the highest percentage of burnout.

Burnout does not occur only in healthcare: The occurrence of burnout syndrome in diverse occupations, e.g., social workers, advisors, teachers, nurses, laboratory workers, speech therapists, police and prison officers, stewardesses, managers, and even in housewives, students, and unemployed people.^24^ In most of these occupations, the combination of caring, advising, healing, or protecting, coupled with the demands of showing that one cares, is of central importance.

In anaesthesiology, PBS manifests in unique ways because of the high-stress, high-stakes nature of the work. Anaesthesiologists often experience emotional exhaustion from prolonged periods of intense concentration and decision-making under pressure. Depersonalization can occur as a coping mechanism against the constant strain of patient care, leading to a sense of detachment or indifference toward patients.^25^ Reduced personal accomplishment in anaesthesiologists may stem from the invisibility of their role; despite being crucial, their work is often behind the scenes and not directly recognized by patients^26^. These manifestations of PBS in anaesthesiology not only impact the mental health of professionals but also potentially affect patient safety and care quality.^6,27^

### Consequences of PBS

Burnout in healthcare has far-reaching consequences, impacting both practitioners and patient care. Burnout symptoms include cognitive challenges like poor concentration and memory lapses.^28^ Personality changes, such as reduced motivation, cynicism, and aggressiveness, are also common.^13^ Physical symptoms include headaches, gastrointestinal issues, and cardiovascular problems like tachycardia and arrhythmia. Socially, burnout results in workplace withdrawal, relationship difficulties, and isolation. In severe cases, it can lead to anxiety, depression, and, tragically, suicide. Healthcare professionals, particularly anaesthesiologists, often develop substance abuse tendencies, turning to alcohol, drugs, and medications. Nearly 10% of them may develop substance-related disorders.^29^ This is because they have access to pharmaceuticals and are usually self-medicating for pain, which can increase the risk of addiction.^30^

However, the consequences of burnout extend beyond the well-being of practitioners. They affect patient care by reducing empathy, increasing medical errors, and diminishing patient satisfaction.^24^

### Burnout Amongst Turkish Anaesthesiologists

Burnout is an issue that Turkish anaesthesiologists are concerned about, as indicated by two studies conducted in the country.^31,32^ The first study aimed to assess the levels of burnout among healthcare workers specializing in Anaesthesiology and Algology in a large Turkish region.^31^ The results were concerning, showing high burnout scores among the participants. Healthcare workers expressed dissatisfaction with working conditions such as environment, working hours and salaries, suggesting that these factors may worsen burnout. The second study focused on trainee anaesthesiologists and shed light on how inexperienced professionals are vulnerable to stress and burnout.^32^ It revealed that perceived stress was significantly high during the years of training, which correlated with increased exhaustion and depersonalization while decreasing personal accomplishment. Additionally, gender and family factors played a role; female anaesthesiologists reported accomplishment and lower depersonalization than their male counterparts.^32^ Trainees with two or more children demonstrated accomplishment while having lower depersonalization and emotional exhaustion scores. These findings emphasize the need to address burnout among anaesthesiologists. The findings from these studies highlight the pressing need to alleviate burnout and promote the physical well-being of anaesthesiologists in Turkey throughout their professional journeys.

### Psychotherapy for Managing Burnout

Psychological treatments play a role in addressing burnout. One effective option is cognitive behavioral therapy (CBT).^33^ CBT is a goal-oriented approach that helps individuals recognize and tackle burnout symptoms. It involves understanding the causes of stress and burnout, adjusting thoughts, enhancing work-related skills, and engaging in leisure activities for recovery.^34^

Studies examining individuals who underwent CBT sessions found reductions in cortisol levels, improvement in well-being, and diminished burnout symptoms.^35^ Another study reported a 64% decrease in burnout and emotional exhaustion following CBT.^36^ Mindfulness, another approach, effectively reduced burnout symptoms.^36^

Acceptance and commitment therapy (ACT) is an intervention that uses acceptance and mindfulness strategies along with commitment and behavior change strategies to increase psychological flexibility. ACT has been shown to lead to a reduction in burnout and its individual subscales.^37,38^ Eye Movement Desensitization and Reprocessing also shows potential for reducing exhaustion among individuals experiencing burnout;^39^ however, additional research is needed for confirmation.

Other forms of therapy, such as music therapy, stress management techniques, spa treatments, and art therapy, have shown potential in reducing the symptoms associated with burnout.^40,41,42^ However, more research is needed to validate these findings.

### Use of Medications to Combat Burnout

Medications, such as antidepressants and sleep aids, are commonly prescribed for individuals experiencing burnout. However, their effectiveness in reducing burnout symptoms remains uncertain. Currently, there is no medication specifically designed for treating burnout. Although psychotropic drugs are used in over half of the cases involving leave due to burnout-related issues,^43^ limited evidence supports their efficacy in treating burnout.

### Strategies for Burnout Prevention

Addressing burnout among anaesthesiologists is a complex challenge, given the demanding nature of their work. Solutions should focus on efficient time management, prioritizing self-care and providing flexible mental health support. Two recent systematic reviews^44,45^ evaluated the effectiveness of interventions in mitigating burnout among physicians. The first review^44^ found that while existing interventions led to small but significant reductions in burnout, organization-directed approaches showed the most promising results. In the second review,^45^ results showed that both individual-focused and structural or organizational strategies could lead to meaningful reductions in burnout levels. However, there was a notable scarcity of organization-directed interventions despite their demonstrated effectiveness. The review emphasized the need for more effective intervention models to combat physician burnout, advocating for approaches that foster healthy relationships between physicians and their work environments.

Both reviews highlight the urgency of addressing physician burnout through multifaceted interventions. While individual-focused strategies can yield positive outcomes, organization-directed approaches offer promising avenues for mitigating burnout and promoting physician well-being on a broader scale. Improving the work environment by incorporating facilities like sports complexes and health-focused cafeterias can promote well-being,^46^ Encouraging outdoor breaks and optimizing workspaces for natural light are also beneficial. Healthcare organizations must offer flexible mental health services, including counseling during non-traditional hours and virtual options.^47^ Destigmatizing mental health is key to ensure anaesthesiologists feel comfortable seeking support^48^. Finally, efficient scheduling practices, such as adequate rest periods and minimizing on-call duties, can help achieve work-life balance.^49^

### Challenges in Treating Physician Burnout

Understanding the challenges in treating physician burnout is crucial for developing effective interventions. A recent study^50^ delved into this issue and revealed two main obstacles:

First, physicians often hesitate to seek help until they reach severe stages of exhaustion. This delay in seeking assistance prolongs their suffering and intensifies burnout symptoms. Additionally, physicians struggle with the role reversal of becoming a patient, making it challenging to accept treatment.

Psychologists attribute these challenges to several factors. Many physicians lack a designated general practitioner, hindering their access to primary care. Moreover, guilt about reducing their workload and difficulty separating their professional and personal lives contribute to their reluctance to seek help.

The study^50^ underscores that these challenges stem from physicians’ perceptions of their professional identity. They view themselves as enduring and selfless, making it difficult to acknowledge their vulnerabilities and prioritize self-care.

## Conclusion

Effectively addressing physician burnout requires a comprehensive strategy that targets both entrenched attitudes and systemic issues within healthcare systems. This necessitates providing accessible and confidential mental health support, advocating for work-life balance, and destigmatizing help-seeking behaviors. We can cultivate a healthier and more resilient healthcare workforce by tackling these challenges head-on.

## Figures and Tables

**Table 1:**
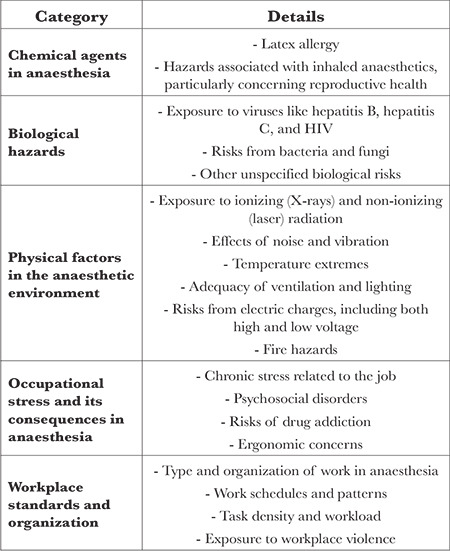
Occupational Health and Safety Risks in Anaesthesia Practice

**Table 2:**
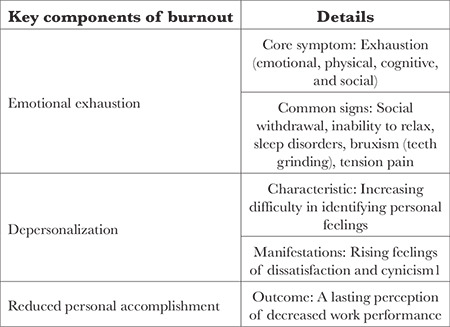
Key Components of Burnout
